# Neuron–Glia Crosstalk and Neuropathic Pain: Involvement in the Modulation of Motor Activity in the Orofacial Region

**DOI:** 10.3390/ijms18102051

**Published:** 2017-09-26

**Authors:** Mohammad Zakir Hossain, Shumpei Unno, Hiroshi Ando, Yuji Masuda, Junichi Kitagawa

**Affiliations:** 1 Department of Oral Physiology, School of Dentistry, Matsumoto Dental University, 1780 Gobara Hirooka, Shiojiri, Nagano 399-0781, Japan; unno_shumpei@po.mdu.ac.jp (S.U.); kitagawa@po.mdu.ac.jp (J.K.); 2 Department of Biology, School of Dentistry, Matsumoto Dental University, 1780 Gobara, Hirooka, Shiojiri, Nagano 399-0781, Japan; andohiroshi@po.mdu.ac.jp; 3 Institute for Oral Science, Matsumoto Dental University, 1780 Gobara, Hirooka, Shiojiri, Nagano 399-0781, Japan; masuday@po.mdu.ac.jp

**Keywords:** satellite glial cells, microglia, astroglia, neuropathic orofacial pain, orofacial motor activity

## Abstract

Neuropathic orofacial pain (NOP) is a debilitating condition. Although the pathophysiology remains unclear, accumulating evidence suggests the involvement of multiple mechanisms in the development of neuropathic pain. Recently, glial cells have been shown to play a key pathogenetic role. Nerve injury leads to an immune response near the site of injury. Satellite glial cells are activated in the peripheral ganglia. Various neural and immune mediators, released at the central terminals of primary afferents, lead to the sensitization of postsynaptic neurons and the activation of glia. The activated glia, in turn, release pro-inflammatory factors, further sensitizing the neurons, and resulting in central sensitization. Recently, we observed the involvement of glia in the alteration of orofacial motor activity in NOP. Microglia and astroglia were activated in the trigeminal sensory and motor nuclei, in parallel with altered motor functions and a decreased pain threshold. A microglial blocker attenuated the reduction in pain threshold, reduced the number of activated microglia, and restored motor activity. We also found an involvement of the astroglial glutamate–glutamine shuttle in the trigeminal motor nucleus in the alteration of the jaw reflex. Neuron–glia crosstalk thus plays an important role in the development of pain and altered motor activity in NOP.

## 1. Introduction

Chronic pain is a major public health problem that has a significant impact on both the individual and community [1,2]. Acute pain is beneficial as it warns against impending or current tissue damage, whereas in contrast, there appear to be no beneficial functions of chronic pain [3]. Neuropathic pain, a type of chronic pain, can result from nerve injury, inflammation, or diseases of the peripheral or central nervous systems, and is characterized by spontaneous pain (ongoing or episodic), pain resulting from stimuli that would not normally provoke pain (allodynia), and exaggerated pain responses to noxious stimuli (hyperalgesia) [3,4]. Neuropathic pain in the head, neck, face, oral or perioral regions is termed neuropathic orofacial pain (NOP) [3,4,5]. The etiology of NOP can include systemic diseases (e.g., diabetes), viral infections (e.g., herpes zoster), nerve compression, and injury to peripheral nerves during dental operative procedures, such as tooth extraction, root canal treatment and dental implant surgery [6,7]. Neuropathic pain is associated with dysfunction throughout the pain pathway, including the nociceptors, the peripheral ganglia, the brainstem or the spinal cord, the thalamus, and the cerebral cortex [8,9,10,11,12]. Neuropathic pain also causes motor impairment or dysfunction [13,14,15]. However, the mechanisms of neuropathic pain are complex, rendering it difficult to treat effectively [8,9,10,11,12].

Recent studies strongly suggest that the activation of glia in the pain transmission pathway plays a critical role in the initiation and maintenance of neuropathic pain [16,17,18,19]. In this review, we discuss the role of glia in the development of neuropathic pain and their involvement in the modulation of orofacial motor activity in the disorder.

## 2. Chronic Orofacial Pain

Chronic orofacial pain is a major health problem, and is associated with high morbidity and health service utilization [20,21]. The prevalence of chronic orofacial pain is unclear, but several studies suggest that it is approximately 7–11% [3,5,22,23,24,25]. Chronic orofacial pain conditions represent a challenge to the clinician because of their complexity. The chronic pain can be musculoskeletal (e.g., temporomandibular disorders, chronic orofacial muscle pain), neuropathic (e.g., trigeminal neuralgia, post-traumatic trigeminal neuropathy, burning mouth syndrome, trigeminal post-herpetic neuralgia, glossopharyngeal neuralgia), vascular (post-stroke pain), or facial pain with headache (e.g., tension type headache, chronic/episodic migraine) [3,5,6,26,27,28,29,30,31,32]. The pain can be episodic (e.g., trigeminal neuralgia) [7,28] or continuous (e.g., burning mouth syndrome, post-traumatic trigeminal neuropathy) [14,29,30]. The chronic pain can also be associated with neuropathy, which is characterized by skin and mucosal numbness in the regions innervated by the trigeminal nerve, and is caused by trauma, autoimmune diseases (e.g., systemic scleroderma, Sjogren’s syndrome and multiple sclerosis), infectious diseases (e.g., syphilis, leprosy and viral infections) or cancer in the orofacial region [2].

## 3. Neuropathic Orofacial Pain

Neuropathic orofacial pain (NOP) is often challenging for dental clinicians to diagnose and treat [2,6]. This condition can arise as the result of injury, inflammation or pathological diseases of either the peripheral or central nervous systems, and is characterized by continuous or episodic pain in orofacial regions [4,8,9,10,11]. This type of pain is often associated with hyperalgesia (exaggerated responses to painful stimuli), allodynia (pain resulting from stimuli that would not normally provoke pain), and abnormal pins-and-needles sensations [4,8,9,10,11]. Numerous clinical entities fall within NOP, including trigeminal neuralgia, post-traumatic trigeminal pain, atypical odontalgia, burning mouth syndrome and glossopharyngeal neuralgia [6,7,28,29,30,31,32]. Trigeminal neuralgia is an episodic neuropathic pain condition, and sufferers often report this pain as a severe, lancinating, electric shock-like pain [7,28]. The pain is often localized in areas innervated by the second and third divisions of the trigeminal nerve, and can be provoked by light touch [7,28]. The most common cause of trigeminal neuralgia is compression of the trigeminal nerve root by an overlying loop of an artery or vein, resulting in demyelination of the trigeminal sensory fibers [7,28]. In trigeminal post-herpetic neuralgia, continuous pain occurs in extraoral and intraoral areas, at the sites of infection of herpes zoster [32]. Burning mouth syndrome is an intraoral NOP condition, which manifests as a continuous burning discomfort of the oral mucosa, especially the tongue [30]. Atypical odontalgia, or persistent dentoalveolar pain, presents as localized pain in the dentoalveolar tissues. The pain may be a dull throbbing continuous pain, and can sometimes be sharp [31]. Post-traumatic trigeminal neuropathy, or peripheral painful traumatic trigeminal neuropathy (PPTTN), can be caused by nerve injury during operative procedures, such as dental extraction, root canal filling, local anaesthetic injection and implant placement, or facial trauma [3,14,29,33,34]. This type of NOP is increasingly common [6], and is characterized by continuous burning, tingling and pins-and-needles-like pain in areas innervated by the trigeminal nerve, including the tooth or tooth-bearing areas [3,14,29]. Patients with neuropathic pain have psychological morbidity and a reduced quality of life [20]. They have reduced ability to work and reduced mobility because of the pain. There is a substantial financial burden on society arising from direct costs for treatment, as well as from indirect costs associated with the loss of the ability to work, the loss of the caregiver’s ability to work, and costs for living assistance [20,21].

## 4. Mechanisms of Neuropathic Orofacial Pain: Glial Involvement

The pathophysiological mechanisms underlying neuropathic pain are not fully understood. Numerous studies suggest that the pathogenesis of neuropathic pain involves multiple complex mechanisms [4,9,10,12]. Many studies on the mechanisms of neuropathic pain have used animal models of injury to the peripheral nerve (e.g., injury to the sciatic nerve, or injury to the trigeminal nerve, such as infraorbital or inferior alveolar nerve injury), which display some characteristic features of neuropathic pain, such as allodynia and hyperalgesia [4,9,10,12]. Injury to the peripheral nerve also causes motor dysfunction. For example, injury to the trigeminal system impairs masticatory performance [6,13,14,15,20]. Multiple sites along the pain pathway are altered after nerve injury [4,9,10,12]. Abnormalities such as spontaneous neural activity and ectopic sensitivity to stimuli develop in the injured and uninjured afferents supplying the affected regions. There are changes in the expression of various molecules in the injured and uninjured afferents, as well as in the ganglia (dorsal root ganglia or trigeminal ganglia), where the cell bodies of afferents are located [4,8,9,10,11,12]. The sensitization of the peripheral nerves leads to central sensitization (sensitization occurs in neurons present in the dorsal horn or in the brainstem trigeminal nuclei), resulting in an augmentation of the response to peripheral stimuli (allodynia or hyperalgesia persist long after the injury to the peripheral nerve) [4,8,9,10,11,12]. Recent studies suggest that the immune response to nerve injury, which is induced both peripherally and centrally, plays an important role in the development and maintenance of neuropathic pain [16,17,18,19]. Resident immune cells and neuroglia are activated, and immune cells from the circulation are recruited in response to nerve injury [16,17,18,19].

**Figure 1:**
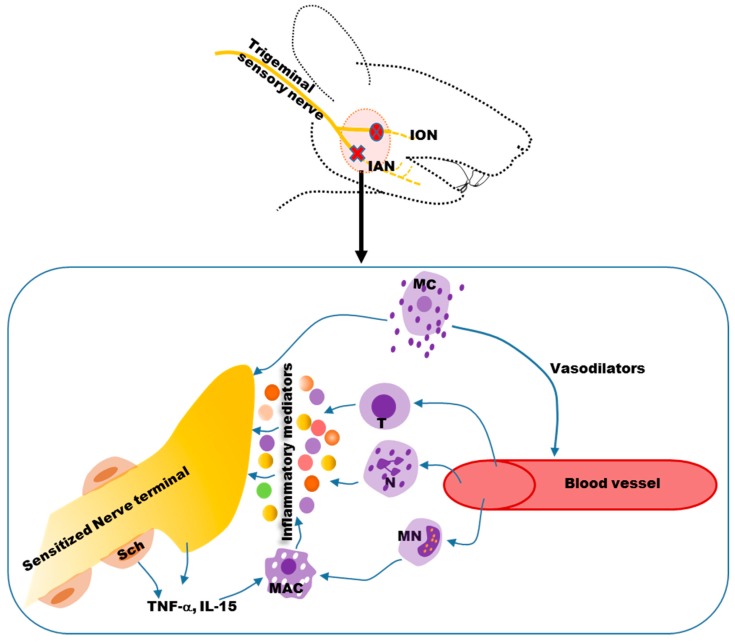
The immune response near the site of a nerve injury sensitizes the nerve terminals. Resident mast cells (MC) are activated and release vasodilators that act on blood vessels, leading to infiltration of immune cells, such as neutrophils, monocytes and T-lymphocytes. Monocytes differentiate into macrophages. These immune cells release inflammatory mediators that sensitize terminals of injured and uninjured nerves. Schwann cells (Sch) that cover the myelinated nerves release cytokines (e.g., TNF-α, IL-15) that also facilitate the recruitment and activation of macrophages. ION: Inferior orbital nerve; IAN: Inferior alveolar nerve; MC: Mast cell; T: T-lymphocyte; N: Neutrophil; MN: Monocyte; MAC: Macrophage; TNF-α: Tumor necrosis factor alpha; IL-15: Interleukin 15.

Peripherally, nerve damage leads to activation of resident mast cells and macrophages and the release of vasodilators (Figure 1), including vasoactive amines and bradykinin [16].

Blood borne immune cells, such as neutrophils, monocytes and T-lymphocytes, infiltrate the site of injury [16]. Inflammatory mediators (Figure 1) are then released from these cells, and act on receptors expressed on nerve terminals, leading to peripheral nociceptor sensitization [17]. In addition, damaged peripheral nerves and Schwann cells release chemokines and cytokines, including tumor necrosis factor alpha (TNF-α), interleukin-15 (IL-15) and interleukin-6 (IL-6), to facilitate the recruitment of macrophages (Figure 1) [18]. The number of macrophages at the site of a spinal nerve injury [35,36] or trigeminal nerve injury [37] is positively correlated with allodynia. IL-6 and nerve growth factors are also increased after infraorbital nerve injury [38].

There is a strong correlation between released cytokines and chemokines at the site of nerve injury and the initiation of neuropathic pain (for a detailed review, see [39,40]). Traditionally, cytokines and chemokines are considered as proteins that regulate the immune response throughout the body. Recent evidences suggest that cytokines and chemokines are released, not only from immune cells, but also from neurons, at the site of the nerve injury. They participate in attracting more immune cells to the site of the injury, to release inflammatory mediators. They also directly act on primary afferents to increase their excitability. Complex interactions occur between various cell types and primary afferent neurons at the site of injury that ultimately result in long-term changes in the excitability of primary afferent neurons [39,40]. A neuropathic pain model, induced by a chronic constriction injury to the sciatic nerve, showed that the cytokines, TNF-α and IL-1β, increased over ten-fold within 1 h in the injured nerve [41]. In addition, inhibiting the action of TNF-α or IL-1β, attenuates the development of chronic pain behavior, like mechanical allodynia and thermal hyperalgesia, in a variety of models of neuropathic pain [42,43,44,45]. Chemokines released at the site of injury also play an important role in the initiation of neuropathic pain. Monocyte chemoattractant protein-1 (MCP-1) or chemokine ligand (CCL) 2 and its receptor, C-C chemokine receptor type 2 (CCR2) are upregulated in the primary afferent neurons and Schwann cells of myelinated nerves in response to the nerve injury [46,47,48,49,50]; in turn, this excites the primary afferents as well as recruits more immune cells to the site of the nerve injury [51]. CCL3 is also upregulated in Schwann cells and in infiltrating macrophages close to injured nerves, and participates in the initiation of neuropathic pain through its receptors CCR1 and CCR5 [40]. Injection of the chemokines—stromal cell-derived factor 1, (SDF1)/C-X-C motif chemokine 12 (CXCL12) and macrophage inflammatory protein 1α (MIP-1α/CCL3)—into the adult rat hind paw produces dose-dependent tactile allodynia [52], believed to be elicited by the activation of chemokine receptors present in the dorsal root ganglion (DRG) neurons [53]. Dorsal root ganglion neurons in culture are also reported to be excited by chemokines [51,54], and the excited neurons release pain-related neurotransmitters, such as substance P and calcitonin gene-related peptide (CGRP) [55,56]. As chemokines can excite primary afferent neurons, and recruit immune cells at the site of nerve injury, they play an important role in simultaneously coordinating inflammation and neuronal excitability [51,52,53,54].

**Figure 2:**
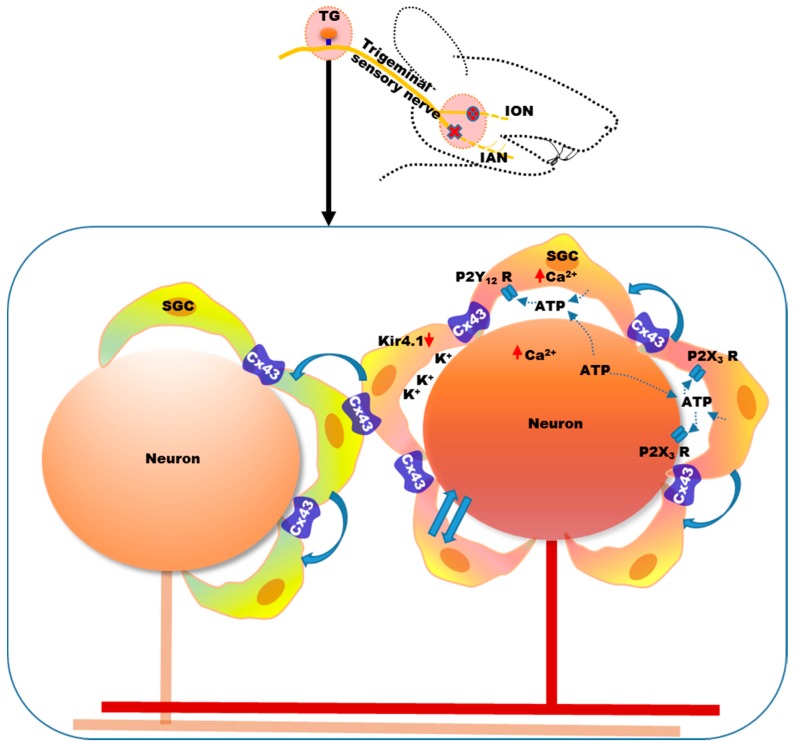
Satellite glial cells (SGCs) surrounding the cell bodies of neurons in the ganglia play an important role in the development of neuropathic pain. Nerve injury leads to the activation and proliferation of SGCs in the sensory ganglia. They interact with neurons through paracrine signaling. ATP, released from SGCs as well as from injured neurons, acts on purinergic receptors, resulting in the mutual activation of neurons and SGCs (indicated in the diagram by straight arrows). Purinergic receptors, P2Y12 and P2X3, are upregulated in the trigeminal ganglion following nerve injury. SGCs express the inwardly rectifying potassium channel, Kir4.1, which helps to maintain extracellular potassium homeostasis. Following nerve injury, expression of Kir4.1 is downregulated in the trigeminal ganglion, thereby increasing extracellular potassium and neuronal excitability. Communication among the SGCs also increases (indicated in the diagram by solid curved arrows), as evidenced by the increase in expression of the common gap junction protein, connexin 43 (Cx43), in the trigeminal ganglion following nerve injury. This communication spreads to the SGCs of nearby neurons, which in turn sensitizes these cells. ATP: Adenosine triphosphate; P2Y12: Purinergic receptor subtype Y12; P2X3: Purinergic receptor subtype X3; Kir4.1: Inwardly rectifying potassium (Kir) channel 4.1. TG: Trigeminal ganglion.

Accumulating evidence suggests that activated glial cells in the sensory ganglia (trigeminal ganglia or dorsal root ganglia) and central nervous system also play a key role in neuropathic pain [16,17,18,19]. Glial cells are non-neuronal cells that provide support and protection for neurons in the central and peripheral nervous systems [57,58,59]. Small satellite glial cells (SGCs) surround the cell bodies of trigeminal ganglion and dorsal root ganglion (DRG) neurons [60]. These cells are connected by gap junctions and are thought to have similar roles to that of astroglia, in the central nervous system [61]. The satellite glial cell marker, glial fibrillary acidic protein (GFAP), increases after nerve injury in the trigeminal ganglion [62,63,64]. SGCs also proliferate in the trigeminal ganglion following a chronic constriction injury of the infraorbital nerve [65]. The gap junction between them increases following trigeminal nerve injury, along with a reduction in the pain threshold [64,66]. Expression of the major gap junction protein, connexin 43 (Cx43), increases in the trigeminal ganglion following inferior alveolar nerve injury, and expression is reduced by application of a selective gap junction blocker (Gap27) to the trigeminal ganglion [64]. These findings suggest increased communication among the SGCs in the trigeminal ganglion following nerve injury [64,66]. The close proximity of SGCs and neuronal cell bodies (Figure 2) favors interactions by paracrine signaling and contributes to the sensitization of afferent neurons [67].

ATP is one of the major transmitters involved in neuron–SGC communication [68]. ATP is released by SGCs and primary afferent neurons, which can increase the intercellular calcium concentration [69,70,71]. ATP plays an important role in communication between SGCs and neurons involving purinergic (P2) receptors (e.g., P2X and P2Y) [69,70,71,72]. Activation of the purinergic receptor, P2Y12R, in SGCs, in the trigeminal ganglion, by ATP, increases calcium influx, which in turn increases the excitability of the cells (Figure 2) [73,74,75]. Injury to the trigeminal nerve, caused by extraction of a tooth, increases purinergic receptor P2X3 and vesicular nucleotide transporter (VNUT) expression in the SGCs and neurons of the trigeminal ganglion, suggesting mutual activation, possibly by VNUT-mediated ATP release [76]. ATP can be released from both trigeminal ganglion neurons and SGCs, which allows them to reciprocally activate each other (Figure 2) [76]. Therefore, increased communication among SGCs, and between neurons and SGCs, after peripheral nerve injury, increases the excitability of primary afferent neurons [68,72].

SGCs in the trigeminal ganglion also play an important role in potassium ion buffering in the ganglion [77]. Extracellular potassium homeostasis is important for maintaining neuronal excitability, and when extracellular potassium is increased, neuronal excitability increases [78]. SGCs express the inward-rectifying potassium channel, Kir4.1, which buffers the potassium concentration in the trigeminal ganglion [77]. The expression of Kir4.1 is downregulated in the trigeminal ganglion (Figure 2) following infraorbital nerve injury, and silencing Kir4.1 with siRNA induces spontaneous and evoked facial pain-like behavior in freely moving rats [79].

The intercellular signaling between SGCs and neurons can spread to neighboring areas, causing cross-excitation within the sensory ganglion, which might underlie extraterritorial pain (Figure 2) [64,80]. It has been observed that injury to the mandibular nerve leads to pain-related cellular changes, not only in neurons and SGCs of the mandibular division, but also in the maxillary and ophthalmic divisions of the trigeminal ganglion [64,80]. Injury to the mandibular nerve also increases the expression of Cx43 in the SGCs surrounding the neurons of the maxillary nerve, suggesting the involvement of SGCs in the development of ectopic hypersensitivity, in the areas innervated by the maxillary nerve, following injury to the mandibular nerve [64].

**Figure 3:**
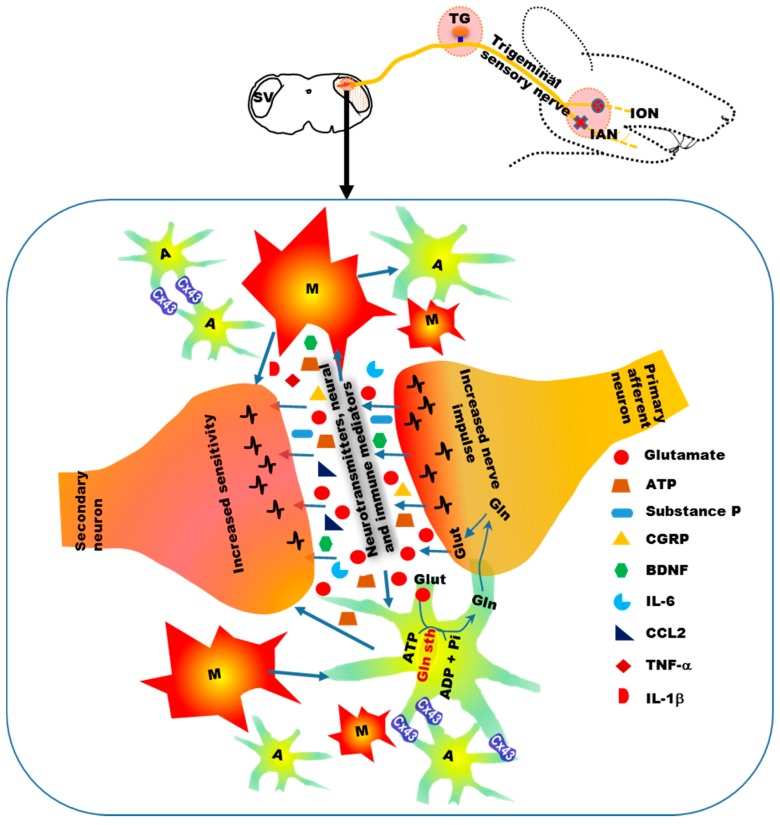
Glial cell involvement in sensory nuclei participates in the development of neuropathic pain. Microglia and astroglia are activated and proliferated in the brainstem sensory nuclei of the trigeminal nerve. Increased primary afferent input following nerve injury causes the release of neurotransmitters and neural and immune mediators, which increase the sensitivity of postsynaptic secondary neurons and activate glial cells. Upon activation, glial cells release mediators (such as ATP, IL-1β, TNF-α and BDNF) that act on secondary neurons and increase their sensitivity. Glutamate–glutamine shuttle activity between astroglia and neurons is increased following nerve injury, thereby increasing the glutamate supply in the synapses between primary and secondary neurons. Cx43 expression increases in astroglia following trigeminal nerve injury, indicating increased communication among the astroglial cells. ATP: Adenosine triphosphate; CGRP: Calcitonin gene-related peptide; BDNF: Brain derived neurotrophic factor; IL-6: Interleukin 6; CCL2: Chemokine ligand 2; IL-1β: Interleukin 1 beta; TNF-α: Tumor necrosis factor alpha; A: Astroglia; M: Microglia.

Along with the peripheral immune response, the immune response in the central nervous system (brainstem trigeminal sensory nuclei, spinal dorsal horn) to peripheral nerve injury also plays a critical role in neuropathic pain [16,18,19]. Microglia serve as the macrophages of the central nervous system, are capable of phagocytosis, and play a role in the repair and scarring processes in the brain and spinal cord, following traumatic injury [57,81]. Increased expression of the microglial markers, Iba1 and OX-42/CD11b, in the central nervous system, following peripheral nerve injury, indicates central activation and proliferation of microglia [82,83,84]. In a model of orofacial neuropathic pain, in which injury is induced to the inferior alveolar nerve by intentional malpositioning of a dental implant during tooth replacement, microglial activation is observed in the brainstem trigeminal subnucleus caudalis [83]. Following nerve injury, microglia in the central nervous system can be activated by increased primary afferent input [85,86], by immune factors from the periphery [18,87], and by infiltrating immune cells from the circulation [88,89]. Increased activity in the primary afferents following nerve injury not only increases postsynaptic secondary neuronal activity, but also activates glial cells in the central nervous system (Figure 3) [85,86]. It has been observed that noxious electrical stimulation of the peripheral nerve increases the expression of the microglial marker, Iba1, in the spinal cord, concomitant with a decrease in pain threshold, indicating that peripheral nerve activity activates central microglia [90,91].

Increased activity of primary afferents following nerve injury causes increased release of neurotransmitters and neural and immune factors, such as glutamate, ATP, substance P, CGRP, brain derived neurotrophic factor (BDNF), IL-6 and CCL2, at central terminals (Figure 3) [16,19,92]. These mediators increase the sensitivity of postsynaptic neurons and activate glial cells surrounding the neurons. Peripherally-released immune factors, such as proinflammatory cytokines (e.g., IL-6), might also activate central glial cells [93,94]. Peripheral IL-6 can be transported to the central nervous system via the circulation, and increase COX-2 activity and PGE2 release in vascular endothelial cells of the brain, leading to a central immune response [93,94].

Infiltration of CD4 (cluster of differentiation 4)-positive T-cells into the spinal cord is observed after spinal nerve transection [88,89]. Infiltration of macrophages or monocytes into the spinal cord occurs following partial sciatic nerve ligation, and these cells can differentiate into microglial-like cells [95]. Astroglia are activated subsequent to microglial activation [16,19,92]. Enhanced neuronal activity after peripheral nerve injury can activate both astroglia and microglia (Figure 3) [16,19,92,96]. Inhibition of nerve injury-induced neuronal activity reduces astroglial marker (GFAP) expression in the spinal cord [96]. Upregulation of GFAP is also observed in the spinal trigeminal complex following application of substance P or CGRP, in an ex vivo medullary slice preparation [18]. In a model of orofacial neuropathic pain induced by inferior alveolar nerve transection, intrathecal administration of fluoroacetate (FA)—an inhibitor of the astroglial metabolic enzyme, aconitase—attenuates nocifensive behavior and suppresses the increase in astroglial activity, suggesting the involvement of astroglia in pain pathogenesis [97]. Moreover, in an orofacial extra-territorial pain model (induced by injury to the upper cervical nerve), GFAP expression is increased in the spinal trigeminal caudalis nucleus, and this increase is suppressed by intrathecal application of FA [98].

Following nerve injury, both microglia and astroglia release chemical mediators that can sensitize neurons in the brainstem trigeminal nucleus and spinal cord [19]. For example, ATP released from nerve terminals and microglia following nerve injury, induces BDNF release from microglia, by activating the purinergic receptor, P2X4. The BDNF binds to its receptor, TrkB, on nociceptive postsynaptic neurons, inducing a shift in the chloride gradient in these cells, which in turn increases their excitability [99,100]. IL-1β, TNF-α and ATP are key mediators, released by activated glial cells, that sensitize neurons [18,101]. IL-1β facilitates *N*-methyl-d-aspartate receptor phosphorylation on neurons, thereby changing their synaptic strength and resulting in enhanced sensitization of neurons, which leads to behavioral hyperalgesia [18,84,102]. Astroglia also interact with neurons to regulate synaptic activity. Glutamate, released at nerve terminals upon the arrival of nerve impulses following nerve injury, activates metabotropic glutamate receptors on astroglia [103]. This leads to the release of various factors by these cells, including glutamine, d-serine and ATP, which in turn modulate neuronal activity [103].

The glutamate–glutamine shuttle between astroglia and neurons also plays a critical role in increasing neuronal excitability after nerve injury (Figure 3) [104,105,106]. Astroglia uptake glutamate that has been released into synapses, which is then synthesized into glutamine by glutamine synthetase [104,105,106]. Neurons use this glutamine to produce glutamate, replenishing the glutamate supply [104,105,106]. The glutamate transporter, GLT-1, is involved in transporting glutamate into astroglia. After nerve injury, GLT-1 is downregulated, resulting in the accumulation of glutamate, thereby increasing the excitability of postsynaptic neurons [107]. In addition, intrathecal application of methionine sulfoximine, an inhibitor of glutamine synthetase, attenuates the elevated excitability of neurons in the spinal trigeminal subnucleus caudalis and medullary dorsal horn, indicating the involvement of the astroglial glutamate–glutamine shuttle in enhancing neuronal excitability [104].

Cx43 expression in astroglia increases following injury (Figure 3) to the trigeminal nerve, and intrathecal administration of a gap junction blocker attenuates the central sensitization of nociceptive neurons in the trigeminal subnucleus caudalis [108,109], indicating increased communication among astroglial cells. Furthermore, activated microglia may also activate astroglia. A recent report suggests that interleukin-18 (IL-18), a pro-inflammatory cytokine, acts as a messenger between microglia and astroglia after peripheral nerve injury [110]. After nerve injury, activated microglia produce IL-18 through the activation of p38 mitogen-activated protein kinases (MAPK). Receptors for IL-18 are also increased on astroglia. The IL-18 signaling leads to the phosphorylation of NF-κB in astroglia, which induces the activation of these cells [110].

## 5. Alteration of Orofacial Motor Activity in Neuropathic Pain: Glial Involvement

Chronic orofacial pain causes psychological and functional impairments [15,20,21]. Chronic pain leads to changes in motor activity [111,112]. Chronic orofacial pain restricts jaw movement, leading to difficulties in orofacial functions, such as mastication, swallowing, speaking and tooth-brushing [15,111]. A study reported a four-fold increase in functional problems—such as difficulty of chewing—in chronic orofacial pain patients, compared with the general population [15]. Limitation of jaw movements (smaller and slower movements) is observed in many experimental pain and clinical pain studies [111,113,114,115]. In animal experiments, noxious stimulation of the orofacial structures reflexively evokes short-duration increases in jaw muscle EMG (electromyogram) activity [116,117]. Injection of mustard oil (a small-fiber excitant and inflammatory irritant) into the temporomandibular joint increases EMG activity in the jaw muscles [117].

**Figure 4:**
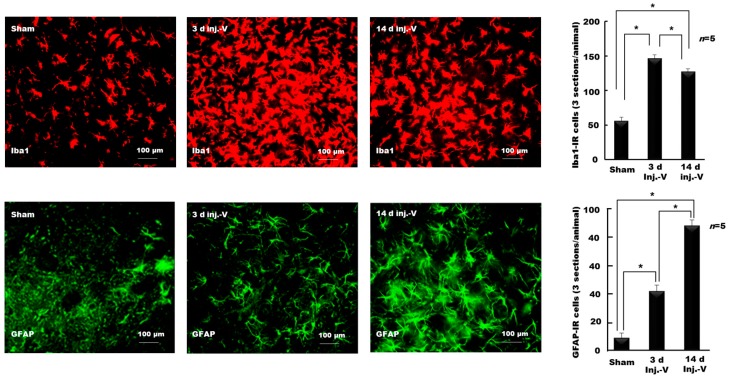
Temporal profile of microglial and astroglial activation in the motor nucleus of the trigeminal nerve following peripheral nerve injury (inj-V: injury to the trigeminal nerve). Following nerve injury, the numbers of activated microglial and astroglial cells (counted on day 3 and 14 following nerve injury) were increased significantly compared with sham-operated rats. The highest number of activated microglia was observed on day 3 following injury; however, the highest number of activated astroglia was observed on day 14 following injury. The figure is modified from our previous published papers [118,119]. * *p* < 0.05.

Recently, we examined whether glia are involved in the alteration of orofacial motor functions in NOP [118,119,120]. We demonstrated that injury to the infraorbital nerve, a sensory branch of the trigeminal nerve, alters masticatory performance in rats [118,119,120]. The time for a complete masticatory sequence (from food intake to the end of the cyclic jaw movements) was longer and the number of complete masticatory sequences was fewer, in nerve-injured rats compared with sham-operated rats [118]. In addition, the time taken to adequately chew food was longer, and the number of chewing cycles was reduced, in nerve-injured rats, compared with sham-operated rats. Furthermore, nerve-injured rats frequently dropped the food. This change in masticatory performance following nerve injury was associated with allodynia (nocifensive behavior) in the facial skin area innervated by the injured nerve and increased expression of the microglial marker, Iba1, in the sensory and motor trigeminal nuclei in the brainstem [118,120]. The highest number of activated microglia was observed on day 3 following injury (Figure 4).

On day 14 following injury, the number of activated microglia was less than that on day 3, but significantly more than in sham-operated rats (Figure 4). Repeated application of a microglial inhibitor (intraperitoneal injection and microinjection into the trigeminal motor nucleus) before and after the nerve injury restored masticatory performance to near pre-injury levels. Minocycline also decreased the expression of microglial markers in the sensory and motor nuclei of the trigeminal nerve, and attenuated nocifensive behavior. Many previous studies reported that microglial blockers (e.g., minocycline) attenuated neuropathic pain, mainly by inhibiting microglial activation and preventing the release of pro-inflammatory cytokines from microglia and other sources [121,122,123,124,125,126,127,128,129,130]. The blockers can inhibit the activation of microglia by inhibiting mitogen-activated protein (MAP) kinase pathways [121,122]. They can inhibit the release of inflammatory mediators, like IL-1β, IL-6, TNFα and NO, from activated microglia [123,124,125]. It might be possible for the microglial blockers to act on microglial receptors (e.g., adrenergic, dopaminergic and cholinergic, adenosine receptors) to inhibit the release of pro-inflammatory mediators from activated microglia in neuropathic pain conditions. They can also inhibit the trafficking of peripheral immune cells into the DRG [126]. Minocycline has been reported to inhibit the expression of major histocompatibility complex 2 (MHC II) on microglia and the subsequent reactivation and infiltration of T lymphocytes into the CNS parenchyma [127,128,129]. Minocycline has also been found to inhibit the downregulation of glial glutamate transporters’ (GTs) expression, following sciatic nerve injury, thereby, preserving the normalized activation of *N*-methyl-d-aspartate (NMDA) receptors in the spinal sensory synapses [130]. The inhibition of microglial activity and attenuation of neuropathic pain behavior by microglial blockers suggests that elevated microglial activity in the sensory and motor nuclei of the trigeminal nerve play a pathogenetic role in orofacial motor dysfunction in neuropathic disease [118,120].

**Figure 5:**
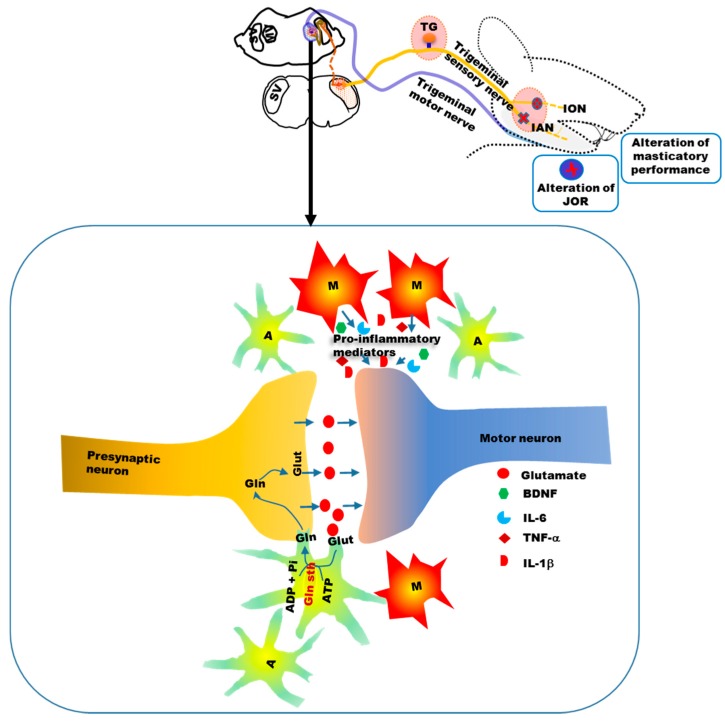
Schematic showing the involvement of glia in the trigeminal motor nucleus in the change in orofacial motor activity following nerve injury. Activated microglia and astroglia are observed in the motor trigeminal nucleus following nerve injury. Similar to sensory nuclei, pro-inflammatory mediators might be released from hyperactive microglia, and these mediators may alter the sensitivity of motor neurons. The astroglial glutamate–glutamine shuttle might also participate in the modulation of motor neuronal activity. BDNF: Brain derived neurotrophic factor; IL-6: Interleukin 6; IL-1β: Interleukin 1 beta; TNF-α: Tumor necrosis factor alpha; Glut: Glutamate; Gln: Glutamine; Gln sth: glutamine synthetase; ADP: Adenosine Diphosphate; Pi: Inorganic phosphate; A: Astroglia; M: Microglia.

A number of previous studies have also shown activation of microglia in the trigeminal sensory nuclei and surrounding areas following injury to the trigeminal nerve [121,131,132]. In addition, injury to the facial and hypoglossal nerves increase microglial activity in the motor nuclei of these nerves [133,134,135]. How activated microglia in the motor nucleus impact motor neuronal excitability is not fully clear. It is possible that, similar to sensory nuclei, pro-inflammatory mediators, released by microglia modulate the excitability of motor neurons, and thereby alter motor functions (Figure 5) [120].

We also observed increased GFAP expression in the trigeminal sensory and motor nuclei following trigeminal peripheral nerve injury [119]. On day 3 following injury, the number of activated astroglia in the trigeminal motor nucleus was significantly more than in sham-operated rats. However, the highest number of activated astroglia was observed on day 14 following injury (Figure 4). Astroglial activation was associated with an increase in the amplitude of the jaw-opening reflex, and allodynia on facial skin. Microinjection of methionine sulfoximine, a blocker of glutamine synthetase, decreased the amplitude of the jaw-opening reflex, which was reversed following microinjection of glutamine into the trigeminal motor nucleus [119]. These findings suggest the involvement of the astroglial glutamate–glutamine shuttle in orofacial motor dysfunction, following trigeminal nerve injury (Figure 5) [119,120]. Hyperactive astroglia may produce more glutamine, which is later converted to glutamate, which would in turn increase motor neuronal excitability (Figure 5). In addition, similar to the sensory nucleus, other factors (e.g, ATP and ILs) released from hyperactive astroglia, may modulate motor neuronal excitability [120]. The temporal patterns of microglial and astroglial cell activation in the motor nucleus of the trigeminal nerve (Figure 4) show that microglia are activated earlier than astroglia following nerve injury, similar to the findings in the sensory nucleus of the trigeminal nerve [16,19,92].

## 6. Conclusions

The etiology and pathophysiology of neuropathic orofacial pain are complex and diverse. Current understanding of the pathophysiology of neuropathic orofacial pain suggests that a sequence of events occurs during the development of neuropathic pain. Nerve injury induces immune responses, both peripherally (around the injured area) and centrally, which play an important role in the development and maintenance of neuropathic pain. Immune cells, glia and neurons form an integrated network that modulates the excitability of pain pathways. Following injury, hyperactive microglia and astroglia in the brainstem trigeminal sensory nuclei participate in the development and maintenance of nocifensive behavior. The development of motor function impairment is an integral event in neuropathic pain conditions. In our recent studies, we observed that hyperactive glial cells in the brainstem trigeminal motor nucleus play a role in the modulation of orofacial motor activity (alteration of masticatory performance and modulation of the jaw reflex). It remains unclear how hyperactive glial cells interact with motor neurons. Pro-inflammatory mediators released from activated glial cells may affect the excitability of motor neurons. Changes in the astroglial glutamate–glutamine shuttle might also be involved in the modulation of the jaw reflex in neuropathic conditions. Future studies on the interaction between glial cells and motor neurons should advance our understanding of the pathogenesis of neuropathic pain.
